# Cholesterol Regulation of Membrane Proteins Revealed by Two-Color Super-Resolution Imaging

**DOI:** 10.3390/membranes13020250

**Published:** 2023-02-20

**Authors:** Zixuan Yuan, Scott B. Hansen

**Affiliations:** 1 Department of Molecular Medicine, Department of Neuroscience, UF Scripps, Jupiter, FL 33458, USA; 2 Department of Neuroscience UF Scripps, Jupiter, FL 33458, USA; 3 Skaggs Graduate School of Chemical and Biological Sciences, The Scripps Research Institute, Jupiter, FL 33458, USA

**Keywords:** ion channel, transporter, PIP_2_, lipid raft, nanoscopic, clustering

## Abstract

Cholesterol and phosphatidyl inositol 4,5-bisphosphate (PIP_2_) are hydrophobic molecules that regulate protein function in the plasma membrane of all cells. In this review, we discuss how changes in cholesterol concentration cause nanoscopic (<200 nm) movements of membrane proteins to regulate their function. Cholesterol is known to cluster many membrane proteins (often palmitoylated proteins) with long-chain saturated lipids. Although PIP_2_ is better known for gating ion channels, in this review, we will discuss a second independent function as a regulator of nanoscopic protein movement that opposes cholesterol clustering. The understanding of the movement of proteins between nanoscopic lipid domains emerged largely through the recent advent of super-resolution imaging and the establishment of two-color techniques to label lipids separate from proteins. We discuss the labeling techniques for imaging, their strengths and weakness, and how they are used to reveal novel mechanisms for an ion channel, transporter, and enzyme function. Among the mechanisms, we describe substrate and ligand presentation and their ability to activate enzymes, gate channels, and transporters rapidly and potently. Finally, we define cholesterol-regulated proteins (CRP) and discuss the role of PIP_2_ in opposing the regulation of cholesterol, as seen through super-resolution imaging.

## 1. Introduction

Elevated cholesterol is a key cellular component important to many metabolic and neurological diseases, including Alzheimer’s, cardio-vasculature, diabetes, viral infections, and chronic inflammation [1,2,3,4,5,6,7,8,9]. A gap in understanding exists for how cholesterol affects many diverse biological functions. As a signaling molecule, cholesterol’s major function is to cluster palmitoylated proteins (proteins containing a 155555 6-carbon lipid) with lipid rafts [10,11,12,13]. The lipid rafts are comprised of saturated ceramide-containing glycolipids (e.g., monosialotetrahexosylganglioside (GM1)) that bind palmitate with high affinity [11]. Clustered GM1 lipids are also referred to as GM1 clusters or GM1 domains.

**Figure 1:**
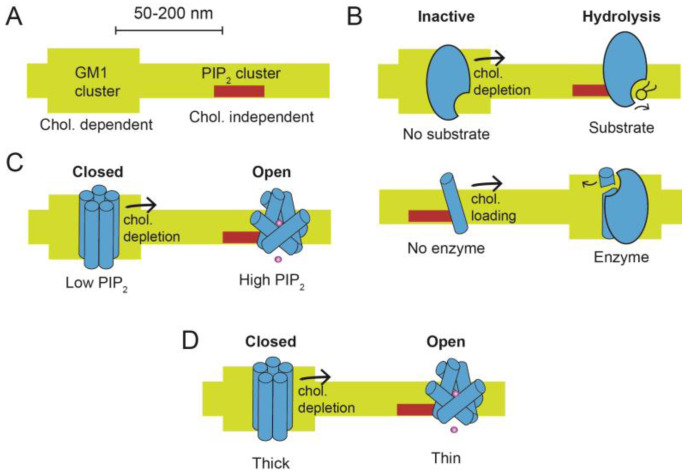
Cholesterol-regulated protein (CRP) activation by cholesterol depletion. (**A**) Two lipid domains are shown. On the left, saturated GM1 lipids are thick and form a cholesterol (chol.) dependent location. On the right, phosphatidylinositol 4,5 bisphosphate (PIP_2_) lipids are clustered and form cholesterol-independent locations. The two locations are separated by 50–200 nm in a cell membrane. (**B**–**D**) CRP proteins move in response to cholesterol depletion. In high cholesterol, proteins reside primarily in the GM1 location. In low cholesterol, they shift to or near the PIP_2_ location. The shift to a new location produces at least three mechanisms of activation. In (**B**), CRP activation is shown by substrate presentation. (Top) An enzyme (blue shading) is inactive when sequestered in a GM1 lipid cluster, away from its substrate. When cholesterol is low, the GM1 domain is disrupted, and the enzyme moves (black arrow) to the PIP_2_ location, where it has access to its substrate. A generic lipid substrate is shown (black stick structure), but the substrate could also be a protein. (Bottom) A protein substrate (blue rod) is shown moving in response to cholesterol loading (black arrow) while the hydrolytic enzyme remains stationary. In (**C**), CRP activation is shown by lipid gating. An ion channel (blue shading) is localized to GM1 clusters, where it is held inactive from a lack of PIP_2_. Disruption of GM1 lipids (black arrow) allows the channel to move to PIP_2_ clusters where PIP_2_ concentration is high. The binding of PIP_2_ causes a conformational change in the transmembrane domain that opens the channel. In (**D**), CRP activation is shown by membrane thickness. An ion channel (blue shading) is sequestered into thick lipids (GM1 lipids). The thickness drives an inactive state. When the channel moves from GM1 lipids, the membrane thins, causing the helices to change conformation and open the channel.

Phosphatidylinositol 4,5 bisphosphate (PIP_2_) is also a membrane-signaling lipid [14,15,16,17,18], and forms charged clusters separate from GM1-containing clusters [19,20,21,22,23] (Figure 1A). The distance between GM1 and PIP_2_ clusters ranges from 42 nm in muscle cells [22] to ~200 nm in lung and kidney cells [7]. PIP_2_ clusters are largely insensitive to cholesterol [19,20,22,23].

Many ion channels, transporters, enzymes, and guanine-coupled protein receptors (GCPRs) are both palmitoylated and contain a PIP_2_ binding site [13,24,25,26]. Where they localize can often influence protein function (Figure 1B–D). Until recently, detecting a protein moving between GM1 and PIP_2_ was precluded by lacking a microscope with sufficient resolution [13].

In the absence of appropriate resolution, the movement of proteins between GM1 and PIP_2_ domains went undetected. Studies of proteins localizing with lipids were limited to biophysical properties. For example, many proteins co-purify with GM1 lipids, and disrupting GM1 clusters alters protein function [27,28,29]. Similarly, PIP_2_ was shown to bind directly to ion channels and transporters using structure determination [15,30,31,32,33,34]. PIP_2_ also dramatically regulates protein function through protein–lipid interactions, including gating ion channels and trafficking membrane proteins [13,14,15,16,17,35,36,37,38,39]. Here we use these biophysical properties and recent findings from super-resolution imaging [7,22,40,41,42,43] to establish basic models for proteins moving nanoscopic distances in the membrane. We define cholesterol-regulated proteins (CRPs) and their activation mechanism by CRP localization. A few recent examples of nanoscopic movement are provided, which may apply to multiple protein families needing further research. To help facilitate more research in this area, we will also discuss the techniques for labeling lipid compartments with super-resolution probes. We also describe the ability of super-resolution imaging to define the nanoscopic topology of membrane proteins with detail appropriate for a non-imaging expert.

## 2. CRP Activation

The movement of proteins’ nanoscopic distances in the membrane is a potent mechanism by which a protein can be activated. A protein moving between lipids has the advantage that the concentration of a lipid substrate or ligand can remain constant in the membrane despite a dramatic change in the concentration experienced by the protein that moves [13,40]. The underlying premise relies only on membrane heterogeneity. Much of the heterogeneity is thought to arise from lipid clustering [10], but there are innumerable ways heterogeneity could arise in the membrane. For the purposes of this review, we will primarily focus on three nanoscopic locations of membrane heterogeneity: GM1 domains, PIP_2_ domains, and disordered lipids. We will also focus on three mechanisms (substrate presentation, ligand presentation, and membrane thickness). We also note that microscopic movement (>250 nm) from the cytosol to the plasma membrane or from the cytosol to the nucleus have parallel mechanisms of substrate presentation; they are briefly mentioned but not discussed.

### 2.1. Substrate Presentation

Substrate presentation is a biological process that employs lipid domains/clusters to activate a protein by selectively exposing the protein to its substrate. In contrast to allosteric activation, the protein need not undergo a conformational change to be activated. The protein simply moves from a region of low substrate concentration to a region of high substrate concentration (Figure 1B). One of the first and best mechanisms for substrate presentation is phospholipase D2 (PLD2) [22]. PLD2 is palmitoylated, and the palmitoylation is responsible for the enzyme’s localization with GM1 clusters and sensitivity to anesthetics [10,44]. The substrate for PLD2, phosphatidylcholine (PC), is primarily poly-unsaturated [45] and resides in the disordered region near PIP_2_. When cholesterol is lowered, PLD2 leaves GM1 clusters and binds to PIP_2,_ where it finds its substrate and produces phosphatidic acid.

In a second type of substrate presentation, the enzyme is permanently clustered, and the substrate moves (Figure 1B, bottom panel). For example, in inflammation and Alzheimer’s disease enzyme activation occurs with the substrate moving into GM1 clusters [46,47,48,49,50,51,52]. Specifically, the amyloid precursor protein (APP) is a substrate for gamma-secretase. The secretase remains clustered to GM1 lipids, and the substrate (APP) moves between GM1 and the disordered membrane region. When cholesterol is high, APP moves to gamma-secretase, where it is cleaved [41].

Classic inflammatory cytokines also work through a substrate movement mechanism. The cytokines are expressed as membrane proteins that reside in the disordered region. During an inflammatory response, cholesterol from the blood is taken up into the cell membrane [3,4]. The cholesterol causes the membrane-bound cytokines to move into GM1 clusters, where they encounter their hydrolytic enzymes. They are cleaved, and the soluble cytokine is released [42,53,54,55,56]. The affinity of the proteins for GM1 lipids is driven by a palmitate-GM1 interaction [11].

In the brain, astrocyte cholesterol regulates the amount of cholesterol available to neighboring cells. For example, astrocytes regulate the ability of neurons to produce amyloid proteins by releasing cholesterol, which is then transported to the neuron and taken up into the plasma membrane [41]. A similar process may occur for regulating cytokine production in microglia [42].

### 2.2. Ligand Presentation

Proteins partitioning into lipids can also rapidly expose a channel or transporter to a lipid ligand. PIP_2_ is chief among the lipid ligands that gate or regulate ion transport proteins [14,15,17]. The same PIP_2_ that localizes a protein to PIP_2_ clusters also serves as a ligand that activates ion transport proteins, including ion channels [15,17,57,58,59,60,61,62,63,64,65]. Hence protein movement from a GM1 domain to a PIP_2_ domain can also gate a channel (See Figure 1C), which we refer to here as “ligand presentation”. A good example of ligand presentation is the channel Twik-related potassium subtype 1 (TREK-1) which depends on its exposure to PIP_2_ and local production of PA in the disordered region for its activation [12,40,66,67]. The lipids bind with ligand-like characteristics that gate the channel [18,68].

Cholesterol can have a similar but opposite effect as PIP_2_ on localization. For example, inward rectifier potassium (K_ir_) channels bind to cholesterol-rich GM1 lipids [69]. But K_ir_ isotype 2.1 (K_ir_2.1) does not always remain in the GM1 clusters; rather, it moves between the two domains [70]. This has at least two consequences [71]. First, cholesterol has a binding site on K_ir_2.1 that is believed to bind cholesterol directly and inhibit the channel [72]. Secondly, however, K_ir_2.1 has a PIP_2_ binding site that gates the channel [30]. As mentioned, GM1 clusters are separated spatially from PIP_2_; hence when the channel is in a GM1 cluster, the concentration of its activating lipid PIP_2_ is decreased. It is unclear which is more important, a lack of PIP_2_ or direct inhibition by cholesterol. However, the combined effect of lipids appears to gate the channel, as the channel is inactive in high cholesterol [73].

In another example, PLD2 binds to TWIK-related potassium channel isotype 1 (TREK-1), producing local PA sufficient to activate the channel [67]. This appears to be an example where substrate presentation combines with lipid gating to activate a channel [66]. The PA binds TREK-1 with µM affinity [68], and local production is estimated in the mM range near the enzyme before diffusion [13].

### 2.3. Membrane Thickness

Ordered lipids are thicker than disordered lipids by up to 10 Å [74,75,76]. When cholesterol increases in the membrane, the ordering of GM1 lipids increases, and the membrane becomes thicker [75,76] (Figure 1A). PIP_2_ clusters reside within the disordered (thin) region of the membrane [20,22,23]; hence moving from GM1 clusters to PIP_2_ clusters requires shifting from thick lipids to thin lipids. The concept of moving between lipids of different thicknesses as a protein activation mechanism was proposed for the gramicidin A channel using artificial membranes [77]. Figure 1D shows a hypothetical channel opening by shifting between membranes of differing thicknesses.

Supporting this model, many channels and transporters are bundles of alpha-helices that span the membrane. When the membrane thins, the helices will either create a hydrophobic mismatch, bend, or lay flat to accommodate the new hydrophobic boundary [78,79]. Structural examples of transmembrane thinning have been observed for bacterial mechanosensitive channels [80,81,82]. For example, early models of large prokaryotic mechanosensitive channel (MscL) openings involved the helices lying flat in the membrane, which allows the pore to expand and dilate [81]. Molecular dynamics studies indicate thinning contributes to the energetics of MscL opening [83].

Recent structural studies with cryoEM show the helices of the small prokaryotic mechanosensitive channel MscS also move horizontally to the plane of the membrane, resulting in a thinner transmembrane section [80,84]. Although bacteria do not have cholesterol, lipid partitioning in bacteria has been considered [85], and membrane thickness drives proteins into lipid compartments that reduce hydrophobic mismatch [86]. Hence, changes in lipid composition or stretch-induced changes in thickness could affect channel opening directly through the membrane.

In the mammalian cell, there are changes in both cholesterol and PIP_2_. The affinity of long transmembrane helices for GM1 lipids increases with increasing membrane thickness [86], which should oppose PIP_2_ affinity. Proteins that change conformation add an independent state to the localization. The concentration of PIP_2_ can also be decreased by flopping to the outer leaflet [39]. Additional research into these potential mechanisms is warranted.

## 3. Combination of Lipid and Protein Labeling Using Two-Color Super-Resolution Imaging

Currently, the best technique to locate a protein within a nanoscopic lipid domain is to label the protein and the lipids and compare their localization using super-resolution imaging. Two-color super-resolution imaging works advantageously with labeled lipids. The lipids are the most stable markers of lipid domain location. Labeling them avoids the uncertainty of a protein marker moving from changes in experimental conditions, particularly changes in cholesterol.

### 3.1. dSTORM

Direct stochastic optical reconstruction microscopy (dSTORM) is a technique that resolves structures below the diffraction limit of light (~250 nM) [87]. To achieve super-resolution, a single fluorophore is measured multiple times, and the center of a gaussian distribution is calculated [88]. The fluorophores used for dSTORM are switchable, i.e., they can be stochastically switched on and off. To measure an isolated fluorophore, only a small number of fluorophores are active at a time [89]. All stochastic methods use some variation of this method to achieve super-resolution.

Lipids can be directly labeled using antibodies and toxins conjugated to appropriate dSTORM-compatible fluorophores. GM1 lipids are typically labeled with fluorescent cholera toxin B (CTxB). PIP_2_ domains are labeled with fluorescent anti-PIP_2_ antibodies. For two-color dSTORM, the proteins are labeled pairwise with the lipids using compatible colors that do not spectrally overlap.

**Figure 2:**
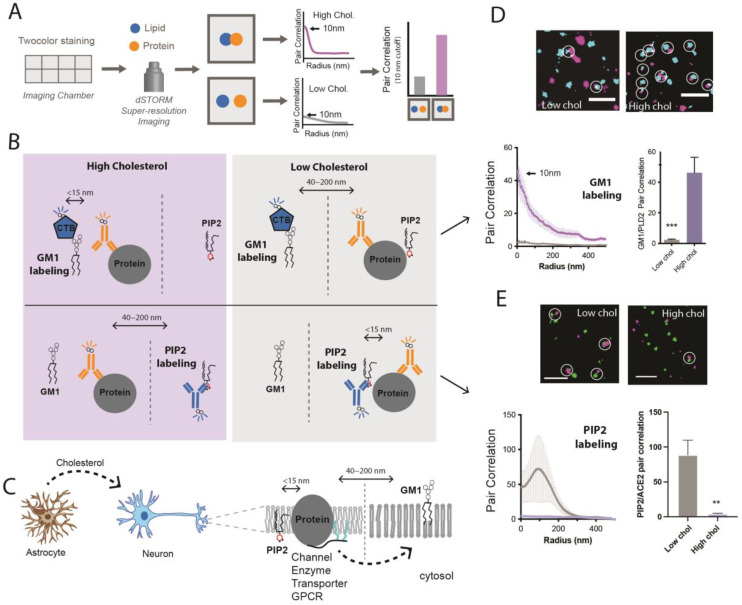
**Strategy for detecting a protein moving between GM1 to PIP_2_ clusters.** (**A**) Labeling scheme and data analysis. Cells grown in 8-well imaging chambers are stained for a lipid and a protein and imaged with super-resolution. Hypothetical pair correlation data (proximity measurement) is shown. High pair correlation at short distances (e.g., 10 nm) indicates an association. (**B**) Strategy for establishing cholesterol-dependent localization. Each lipid compartment is labeled pairwise with protein in either high or low cholesterol. Typically, GM1 clusters and proteins are fluorescently labeled with Cholera Toxin B or antibody. (**C**) Model for CRP regulation in a neuron by astrocyte cholesterol. Astrocytes produce cholesterol which is transported to neurons with apolipoprotein E (ApoE). The neuron takes up the cholesterol into the plasma membrane, where it sequesters proteins with ordered GM1 lipids. (**D**) Pair correlation data of phospholipase D2 leaving a lipid raft after treating cells to remove cholesterol. Data adapted from Pavel et al., PNAS 2020 [40]. (**E**) Pair correlation data of angiotensin-converting enzyme 2 (ACE2) moving to PIP_2_ domains after cholesterol removal. Adapted from Wang et al., 2020 [6]. ** *p* < 0.01 and *** *p* < 0.001.

Figure 2A,B shows a strategy for monitoring the nanoscopic movement of proteins in a membrane. The technique works with cells or whole tissue [7,22,41,90]. For cultured cells, the tissue is grown in an imaging chamber. The cells are fixed, and the lipids and proteins are fluorescently labeled (Figure 2A,B).

Super-resolution imaging uses pair correlation to associate a protein with a lipid domain. Pair correlation in super-resolution imaging is similar to “co-localization” in traditional confocal microscopy. Super-resolution microscopes identify the location of individual molecules. A radius is calculated between molecules, and the molecules with pair correlation at the shortest radii are considered “co-localized”.

Typically, a condition is directly compared to a control, and one determines a ‘shift’ in co-localization. This is particularly convincing when a protein decreases its co-localization with GM1 lipids and increases its co-localization with PIP_2_ [6,22]. This shows a shift from GM1 to PIP_2_ lipids (Figure 2C–E).

The starting location of a protein is critical for understanding if it has moved or not. In most cases, the proteins need to be endogenously expressed. Over-expressed proteins can saturate the GM1 lipids and spill into the regions they would not otherwise occupy without activation. For example, over-expression of PLD dramatically activates TREK-1 [66,67]—presumably by spilling out into the disordered region where both the enzyme and TREK-1 are activated [22,66,91]. For obvious reasons, localizing a protein to the wrong membrane compartment could be determinantal to the purpose of the experiment. For example, the over-expression of ACE2 alters the interaction of SARS-CoV-2 with its hydrolytic enzymes [92]. In theory, over-expressed proteins can yield data directly opposite of reality. Data from endogenously expressed proteins are generally the most reliable.

### 3.2. Understanding Cholesterol’s Role in Disease

Cholesterol’s concentration in the membrane of cells can be adjusted up or down to determine whether a particular protein’s association with a GM1 lipid is sensitive to cholesterol. As mentioned, cholesterol causes increased nematic order in the GM1 lipids. The order reduces fluidity in the ordered region. However, in the disordered region, cholesterol acts as a lubricant that untangles acyl chains and allows them to be more fluid [93]. In general, cholesterol combines to fluidize the membrane [10]. The effect of a protein moving to lipids and the fluidity changing are likely synergistic, but sorting out the relative contributions to disease has been challenging. One of the biggest challenges is simply adjusting the cholesterol level in the cell membrane.

In the brain, astrocytes use apoE to control cholesterol loading into neurons [41,42]. The same apoE can be purified and added to cell cultures with and without a source of cholesterol to respectively load and unload cholesterol into cells [6,7,41,42]. PLD2 and ACE2 are an example of enzymes that move in and out of GM1 domains in a cholesterol-dependent manner (Figure 2D,E). A model for brain cholesterol-regulating protein localization is shown in Figure 2C.

### 3.3. PALM

Photo-activated localization microscopy (PALM) super-resolution imaging is a similar technique to dSTORM, which uses photoactivatable proteins. Using proteins allows for genetically encoding the fluorescent label needed for super-resolution [94]. Genetically encoded PIP_2_ sensors are available for PALM imaging [43,95]. PIP_2_ is typically measured on the inner leaflet of the plasma membrane, which is not accessible in live cells. Genetically encoded PIP_2_ sensors have the advantage of visualizing the proteins without membrane permeabilization. Even in fixed cells, membrane permeabilization could be problematic since the membrane is what one intends to observe.

The genetically encoded PIP_2_ sensors are comprised of the PLC-δ PIP_2_ binding domain and a genetically encoded PALM protein. This technique was recently used to show the clustering of SARS-CoV-2 (SARS2) spike protein with PIP_2_ [43,96]. The spike protein was genetically encoded with dendra2 and the PIP_2_ binding domain from PLC-δ with PAmKate. Similar to dSTORM, the proteins have non-overlapping spectra, and they are suitable for cross-pair correlation analysis.

Similar to the PALM study, SARS-CoV-2 viral entry was studied with dSTORM looking at the viral receptor angiotensin-converting enzyme 2 (ACE2) co-localization with PIP_2_ [7]. Since the spike protein binds to the ACE2 receptor, the two studies provide the best comparison of dSTORM in fixed cells with PALM in live cells. Using live cell PALM, the PIP_2_ cluster sizes appeared larger than in similar studies done in fixed cells [7,95]. Nonetheless, both studies confirm the power of super-resolution imaging to visualize the nanoscopic location of viral entry.

## 4. The Palmitate Binding Site

The affinity of proteins to GM1 lipids depends on palmitate binding to saturated ceramides of GM1 lipids [11]. Kai Simons produced early data showing a protein’s affinity for lipids using detergent-resistant membranes [44]. The Tsien lab used palmitoylation combined with genetically encoded FRET partners to detect movement in and out of GM1 clusters in a biological membrane [96].

**Figure 3:**
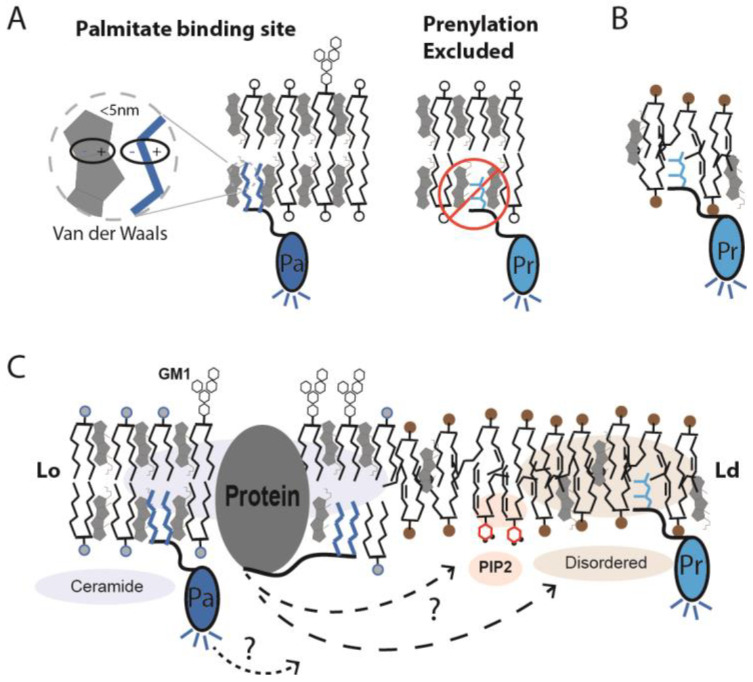
**Palmitate binding site in ordered lipids.** (**A**) A model depicting the molecular basis for GM1 lipids selectively binding to palmitoylated protein (Pa). GM1 lipids are saturated and pack well with cholesterol and palmitate. The site is specific and does not bind prenylated (Pr) proteins. The branched structure of prenyl lipids is thought to clash with the structured palmitate site, thus excluding prenylated proteins from ordered lipid domains (Lo). When attached to a fluorescent protein (dark blue shaded protein), the palmitoylation becomes a sensor for the order of GM1 lipids. (**B**) The prenylation binding site is shown within disordered lipids (unsaturated with bent acyl chains). When coupled to a genetically encoded fluorescent protein (light blue shading), the prenylated protein functions as a sensor of prenyl localization. (**C**) In cellular membranes, the covalent attachment of a palmitate or a prenyl lipid to a protein tags the protein for sorting between GM1 and disordered lipids, respectively. If the protein is a fluorescent protein, the respective compartments are fluorescently labeled. (**B**) When the order of the GM1 lipids is decreased, the palmitate binding site is disrupted, and the palmitoylated proteins can move away from the GM1 lipids. Some proteins move to PIP_2_ clusters (red shading), some move to disordered lipids (tan shading), and some proteins may remain clustered with GM1. Without labeling the lipid, the movement of proteins between lipids is unclear, as depicted by a ‘?’ in the figure.

The mechanism of palmitate affinity is based on nematic order [11,97,98,99]. Like proteins, lipids have regions of order and disorder [10]. The ordered GM1 structures are formed from saturated ceramides that orient perpendicular to the surface of the membrane. Once ordered, they provide an energetically favorable binding surface/pocket for palmitate (Figure 3A). Palmitate is a saturated 16-carbon lipid. When extended, the saturated carbons match the surface and maximize Van der Waals interactions (Figure 3A). In cells, the lipid is naturally covalently attached to proteins in a process called palmitoylation. The palmitoylated proteins are localized to GM1 lipids by the lipid-lipid interaction. A covalently attached myristate (14-carbon saturated lipid) has a similar effect [100].

Prenylation is a second type of lipid modification that attaches a branched unsaturated lipid. The branching is incompatible with the rigid flat surface of the cholesterol. Hence, a protein with a prenyl group is excluded from the ordered GM1 domain. This was first shown by FRET [96] and, more recently, with super-resolution imaging [90].

The selectivity of the palmitate binding site is remarkable and experimentally demonstrates that the order is present in the lipids. If the cell were homogenous, there would be no separation of the two lipid modifications. In the cell, these post-translational modifications serve as localization tags that separate proteins. For additional understanding of lipidation, we refer the reader to basic reviews on palmitoylation [100,101] and prenylation [102,103].

Lipid order is not to be confused with lipid partitioning. Lipid partitioning separates saturated and unsaturated lipids to form separate domains, but the partitioning does not necessarily create order within the domains. Lipid order is dictated by the structure of the lipids and proteins within a particular domain. The ordered palmitate binding site exists in pure saturated lipids, absent any partitioning.

The palmitoylated probes are suitable for studying the state or function of the GM1 cluster. Through understanding the function of the domain, one can begin to predict how the palmitoylation affects the association of a protein with GM1 clusters. However, the palmitoylated probes do not typically reveal how individual proteins function in the GM1 clusters. The reason being many proteins have both palmitoylation and PIP_2_ binding sites. Hence, a protein can shift between the domains based on PIP_2_ levels independent of cholesterol’s effect on palmitoylation. Furthermore, some proteins may not be palmitoylated; rather, they bind to a palmitoylated protein. For example, TREK-1 is not palmitoylated, but it binds phospholipase D2, which is palmitoylated [22,67].

The thickness of a protein also directs its localization [86]. And as mentioned previously, proteins undergo conformational changes that affect their membrane thickness. Cleavage of a transmembrane helix in the membrane can also affect its thickness. Hence, the location of a protein depends on many factors independent of palmitoylation. Thus, a palmitoylation probe alone is insufficient for predicting how a protein will interact with GM1 clusters. Furthermore, the palmitoylation and prenylation probes do not indicate a direct association with PIP_2_ or other lipids that may cluster proteins.

## 5. Strengths and Weaknesses of Imaging Techniques

Directly labeling proteins and lipids has advantages over lipidated probes. When the cholesterol is lowered, palmitoylated proteins tend to leave lipid rafts [6,7,41]. Hence, a probe will leave and no longer report the localization of a protein with a GM1 domain. This is a problem for both the FRET probes and the dSTORM probes described previously. For example, when PLD2 disassociates with a GM1 cluster, it could end up in the disordered region with the probe, which could cause confusion. If the protein happens to bind PIP_2_, the probe and the protein will be separated, but only because the probe does not bind PIP_2_, not because the probe is marking the GM1 domain (Figure 3C).

As additional proteins are identified that remain in the lipid rafts, those proteins may serve as resident markers. For example, gamma-secretase is cholesterol independent and appears to remain in lipid rafts under any condition. In contrast, its substrate APP moves in and out of lipid rafts as astrocytes raise and lower (respectively) the cholesterol [41]. Other proteins like clathrin and flotillin are also very good candidates for marking the lipid compartments. However, there will always be the risk that, in a new condition, a protein marker leaves the lipid raft. The only way to know the lipid location for sure will be to label the endogenous lipids and the protein of interest.

**Figure 4:**
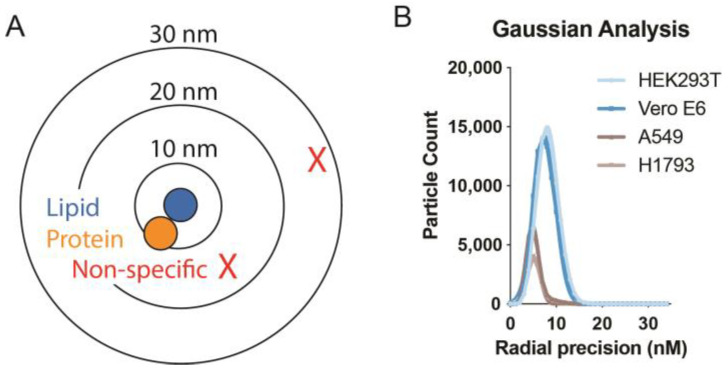
**Non-specific labeling in two-color dSTORM.** (**A**) The bin size in radius (10, 20, and 30nm) is drawn at scale. Proteins (orange, <5 nm in diameter) can associate with a lipid (blue shading). Non-specific labeling (red X) occurs randomly and does not typically associate directly with the lipid or protein. When a radius of 5–10 nm is used, the random background is excluded from the measurement by virtue of proximity. The shorter the distance, the more background is excluded allowing pair correlation for low abundant proteins or those with a high background. (**B**) Example of precision measurements from a dSTORM super-resolution instrument (Vutara VXL). The cells were stained for GM1 and PIP_2_ lipids. Radial precisions in 4 different cell types varied between 4 and 8 nm. Hence very little random overlap occurs due to precision above 10 nm. Taken from Yuan et al., 2022 with permission [7].

A background reduction is one advantage of super-resolution imaging. The background is lowered by quantitating the labeling only in the proximity of the protein of interest (Figure 4A). Often endogenous proteins are expressed at very low levels. A low signal inevitably leads to high background. With super-resolution imaging, noise is excluded by looking at very short distances. Proteins are around 5 nm in diameter. A protein bound directly to another protein should be within 5–10 nm. Proteins that are 50 and 100 nm away are not co-localized. The probability that background labeling is within 5–10 nm is very low under normal protein densities (see Figure 4A for an illustration). Hence, the random association of background at short distances is only significant when the background concentration is very high.

The precision of the dSTORM instrument is around 5–10 nm depending on the cell type and the labeling. Figure 4B shows dSTORM precision data from 4 cell types (data taken from previous publications under open source license [7]). In practice, a precision of 5–10 nm is a sufficiently short radius so that background observations have a reduced impact on results from pair correlation analysis.

One weakness of the PALM method is the over-expression of the PIP_2_ sensor. The sensor has high affinity and logically should compete with proteins that transiently bind to PIP_2_. Theoretically, this should drive PIP_2_ binding proteins to associate more with GM1 lipids. A second weakness is the over-expression of the proteins of interest. Over-expression can saturate the lipid raft and cause the over-expressed protein to spill into an unregulated region. For example, phospholipase D2 (PLD2), which is inactivated by sequestration into lipid rafts, is activated by over-expression. Over-expression alone significantly increases PLD2 activity and TREK-1 currents [67,104]. Expressing genetically encoded proteins under endogenous promoters will alleviate this problem.

Some consideration needs to be given to artificial clustering by antibodies and CTxB. Even in fixed cells, lipids appear mobile over short distances, especially saturated lipids [105]. For lipids like GM1 that cluster on their own, pentadentate binding of the sugar head groups can lead to additional clustering. The clustering affects the cluster size but does not appear to contribute much to function. As mentioned, the function of GM1 lipids is based on the binding palmitate, which is dictated by the ordering of the lipid, not the size of the cluster per se. This is evident from anesthetic treatments which disrupt the order while increasing the cluster size [40]. At microscopic distances, measured by fluorescence recovery after photobleaching (FRAP), cholera toxin-labeled GM1 lipids appear relatively immobile [12].

**Figure 5:**
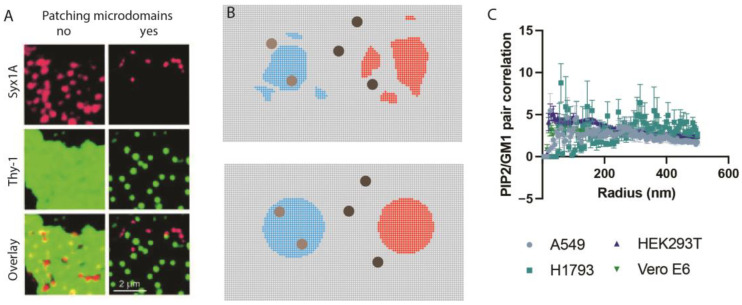
**Antibody patching.** (**A**) antibody patching in unfixed cells. Patches can be seen without super-resolution imaging. Adapted from Lang et al., 2001 with permission [107]. (**B**) Depiction of antibody patching at nanoscopic levels. In the top panel, there are multiple patches of different sizes. In the bottom panel, clustering helps create lipid domains of more uniform size which is important to the cluster analysis. In light blue, the clustering pulls together proteins more associated with the lipid domains. In red, the clustering separates proteins less associated with the lipid domains. (**C**) Pair correlation of PIP_2_ with GM1 in multiple cell types. Pair correlation is very low, suggesting PIP_2_ and GM1 lipids are separate. A549 are lung epithelial, Human embryonic kidney (HEK293), Vero E6 monkey kidney, and H1793 lung epithelial. Data adapted from Yuan et al., 2022 with permission [7].

Some artificial clustering, especially at short distances, can improve pair correlation by creating more distinct clusters. Prior to super-resolution imaging, Reinhard Jahn developed a technique called antibody patching [106] to determine the co-localization of proteins. Antibody patching uses antibodies to create protein clusters discernable by a confocal microscope. Proteins that were associated were driven into the same clusters, and proteins that were not associated were separated by the clustering (see Figure 5A, data reproduced with permission).

In theory, the antibody patch principle should work for super-resolution microscopy. Figure 5B shows a hypothetical enhanced clustering of GM1 and PIP_2_. The proteins are first fixed and then stained with cholera toxin B (CTxB). This allows for limited artificial clustering of the saturated lipids (the marker) while avoiding artificial clustering of palmitoylated proteins (the protein of interest). From experimentation, the average lipid cluster size is typically <200 nm after fixing [7,22,40,41,42], demonstrating that any clustering of lipids is limited to nanoscopic distances.

## 6. Future Directions

Going forward, nanoscopic topology and its dynamic function must be considered for all classes of palmitoylated and PIP_2_ binding proteins. As mentioned, many classes of ion channels and transporters are palmitoylated [24], and many channels and transporters are regulated by PIP_2_. GPCRs have palmytoilation in all their alpha subunits, prenylation in all their G-beta gamma subunits, and a putative PIP_2_ binding site in the GPCR [26]. Their role in CRP activation is largely unknown.

Labels for additional compartments also need to be developed. PIP_3_ appears to reside separately from PIP_2_ and GM1 [23]. Lipid-gated Ion channels and several transporters respond differently to PIP_2_ and PIP_3_ [15]. The relative concentrations are important as PTEN is an enzyme that converts PIP_3_ to PIP_2,_ and mutation contributes to autism [107].

It is unclear whether PIP_3_ partitions into a separate compartment or is maintained separately through its local production and degradation. Early mass spec studies suggested that PIP_3_ contains short-chain saturated lipids vs. long-chain unsaturation in PIP_2_ [15]. Hence the lipids could partition based on lipid hydrophobicity. However, the separation could be generated independently of lipid partitioning or by flopping to the outer leaflet [39]. One speculative mechanism might involve local production near an enzyme. As the lipid is generated, it will be highest near its metabolic enzymes.

The separation of PIP_2_ and GM1 is still somewhat controversial. Early studies found PIP_2_ in pull-down experiments with detergent-resistant membranes (DRMs) [108]. This led investigators to conclude that PIP_2_ resides in lipid rafts. However, the association of PIP_2_ to GM1 lipids through proteins that bind both lipids was not considered. Some percentage of DRM proteins will have PIP_2_ bound, and if there are lots of proteins that bind both PIP_2_ and GM1, there could be a significant amount of PIP_2_ associated with DRMs. Early indirect experiments support a distinct location for PIP_2_ [109].

The most reliable experiment for testing PIP_2_ and GM1 co-localization is super-resolution imaging in the intact membrane. Three groups labeled the membrane and found that GM1 and PIP_2_ appeared separated. Figure 5C shows pair correlation from 4 cell lines with little to no pair correlation. No group using super-resolution imaging has concluded that significant PIP_2_ resides in GM1 clusters. Furthermore, PIP_2_ is arachidonylated, so there is no chemical basis for PIP_2_ being associated with ordered GM1 lipids. PIP_2_ should be excluded from lipid rafts for the same reasons prenylated proteins are excluded. As more proteins emerge that use movement between lipid rafts and PIP_2_ as their activation mode, this GM1/PIP_2_ paradigm will continue solidifying. While speculative at this point, some proteins that have very high affinities for PIP_2_ may attract PIP_2_ into GM1 clusters. This would tend to disrupt the lipid order, but would not necessarily be true of detergent-resistant membranes. Once the membrane is disrupted by detergent, proteins associated with GM1 lipids may also bind the dispersed PIP_2_.

In conclusion, the extent of CRP localization is scarcely known. The number of proteins regulated by cholesterol and nanoscopic movement is likely extensive and integral to many diseases where cholesterol levels are known to be important. Having the right tools to investigate the mechanisms is critical and will likely continue to improve as the technology expands through the ion transport and enzyme communities.

## Data Availability

Not applicable.
